# Two-dimensional phos-tag zymograms for tracing phosphoproteins by activity in-gel staining

**DOI:** 10.3389/fpls.2015.00230

**Published:** 2015-04-14

**Authors:** Claudia-Nicole Meisrimler, Alexandra Schwendke, Sabine Lüthje

**Affiliations:** ^1^Plant Physiology, Biocenter Klein Flottbek and Botanical Garden, University of HamburgHamburg, Germany; ^2^Laboratoire de Biologie du Développement des Plantes, Commissariat à l'Energie Atomique et aux Energies Alternatives, Institut de Biologie, Environnementale et de BiotechnologieSaint-Paul-lez-Durance, France

**Keywords:** phosphorylation, IEF, phos-tag, NEPHGE, high resolution clear native electrophoresis, in-gel activity staining

## Abstract

Protein phosphorylation is one of the most common post-translational modifications regulating many cellular processes. The phos-tag technology was combined with two-dimensional zymograms, which consisted of non-reducing IEF PAGE or NEPHGE in the first dimension and high resolution clear native electrophoresis (hrCNE) in the second dimension. The combination of these electrophoresis methods was mild enough to accomplish in-gel activity staining for Fe(III)-reductases by NADH/Fe(III)-citrate/ferrozine, 3,3′-Diaminobenzidine/H_2_O_2_ or TMB/H_2_O_2_ in the second dimension. The phos-tag zymograms can be used to investigate phosphorylation-dependent changes in enzyme activity. Phos-tag zymograms can be combined with further downstream analysis like mass spectrometry. Non-reducing IEF will resolve proteins with a pI of 3–10, whereas non-reducing NEPHGE finds application for alkaline proteins with a pI higher than eight. Advantages and disadvantages of these new methods will be discussed in detail.

## Introduction

Protein phosphorylation, one of the most common post-translational modifications, can alter enzyme activity and subcellular localization as well as target proteins for degradation and can effect changes in protein-protein interactions (Cousin et al., 2013; Gerbeth et al., 2013; Uhrig et al., 2013). Monitoring the phosphorylation status of proteins is, thus, very important for the evaluation of diverse biological processes. Methods to quantify particular phosphorylation events include radioactive labeling, immunodetection of site-specific phosphorylations, phospho-specific site mapping in peptide mass fingerprinting, chemical labeling but also in-gel phospho stainings (e.g., Pro-Q Diamond®, all blue and quercetin staining) (Ferrão et al., 2012; Wang et al., 2014).

Complex protein samples are often separated by polyacrylamide gel electrophoresis (PAGE), before mass spectrometry (MS) analysis. After PAGE, immunodetection or phospho staining are the most commonly applied techniques to detect phosphorylated proteins. The fluorescent stain, Pro-Q Diamond® by life technologies, gives the opportunity to detect phosphoserine-, phosphothreonine-, and phosphotyrosine-containing proteins without sequence or context specificity (Miller et al., 2006). Currently Pro-Q Diamond® is a standard staining for SDS-PAGE. In contrast it is not often combined with native PAGE (Tsunaka et al., 2009) and no literature can be found on the combination with zymograms. An alternative to phospho staining is phos-tag PAGE, a phosphate affinity electrophoresis for the mobility shift of phosphoproteins (Kinoshita et al., 2006; Kinoshita-Kikuta et al., 2007; Kinoshita and Kinoshita-Kikuta, 2012). A dinuclear metal [Mn(II) or Zn(II)] complex of 1,3-bis[bis(pyridin-2-ylmethyl)-amino]propan-2-olato acts as a phosphate-binding tag molecule, phos-tag, in an aqueous solution under physiological conditions. Recently Mn(II)-phos-tag Blue Native PAGE (BNE) was accomplished in the first dimension (Deswal et al., 2010).

Native PAGE methods in combination with phosphorylation analysis are mainly needed for the characterization of protein-protein interactions, complex assembly and activity regulation, which are prerequisite for the understanding of cellular processes. A variety of native PAGE methods exist (BNE, CNE, native Tris-PAGE, native Acetate-PAGE) and the most optimal can be chosen depending on the sample and the scientific question to be answered. Native PAGEs, with more or less modified protocols, are often used for zymograms because of reduced denaturizing conditions, e.g., high salt concentrations, reducing agents, and strong detergents (Wittig and Schägger, 2005, 2008; Wittig et al., 2006, 2007; Burré et al., 2009; Führs et al., 2009, 2010) which can effect the activity of a protein. It is likely that strong detergents (e.g., SDS), reductants [dithiothreitol (DTT), 2-mercaptoethanol] or heating could not only influence the protein activity but also the phosphorylation.

The standard zymograms (non-reducing SDS-PAGE, without heating of the protein sample) are commonly used for proteolytic enzymes (Vandooren et al., 2013), but combination of different electrophoresis methods and various in-gel activity staining makes the method applicable for different enzyme activities (Manchenko, 2002). In the past, activity in-gel stainings after isoelectric focusing (IEF) slab gels were reported for different enzymes, e.g., malate dehydrogase, peroxidase, quinone reductase, Fe(III)-reductase, superoxide dismutase, catalase and others (Mika et al., 2010; Meisrimler et al., 2011; Kukavica et al., 2012; Lüthje et al., 2014). In the standard IEF-PAGE, protein separation is based on their pI and oriented from basic to acidic pH. A related method, the non-equilibrium pH gel electrophoresis (NEPHGE), also separates proteins by their pI. In NEPHGE, the protein separation is reversed in comparison to IEF-PAGE. NEPHGE was developed to resolve proteins with extremely basic pI (pH 8.5–12.0) (Lopez, 2002). During NEPHGE, proteins are not focused to their pI as in the standard IEF-PAGE. Instead proteins move through the gel based on their charge. For this reason, the accumulated volt hours (Vh) determine the protein pattern across the gel and have to be kept constant to ensure reproducibility.

Furthermore, different native PAGE procedures, e.g. blue native PAGE (BNE), high resolution clear native PAGE (hrCNE) (Wittig et al., 2006, 2007; Wittig and Schägger, 2008; Burré et al., 2009) and native Tris-PAGE (Weydert and Cullen, 2010) were used in combination with in-gel activity staining.

To date, native PAGE methods, as the described above, are well-established systems but none of them is usually named a standard method. Especially the combination of non-reducing IEF/NEPHGE with one or the other native PAGE in the second dimension has rarely been performed and is scarcely found in the literature, but has been important for two-dimensional zymograms (Lüthje et al., 2014). After multiple modifications and trials we now developed a protocol which we report in the present paper. It offers good resolution for the combination of non-reducing IEF or NEPHGE in the first dimension with hrCNE in the second dimension. The hrCNE was combined with the phos-tag to separate proteins depending on their phosphorylation. Various activity in-gel stainings can be accomplished in the first dimension and in the second dimension hrCNE or phos-tag hrCNE. For the first time, we attempted to directly link the phosphorylation status of an enzyme to its activity using 2D zymograms by combining several gel electrophoresis methods based on size, charge and affinity.

## Materials and methods

### Plant material

Proteins were obtained from leaves of 4 week old corn plants (*Zea mays* L. cv. Goldener Badischer Landmais, Saatenunion, Hannover, Germany) and roots of 19 day old pea (*Pisum sativum* L.) plants (Sperli cv. vroege, Lüneburg, Germany). Soluble proteins of corn and pea were separated by differential centrifugation from the microsomal fraction as described elsewhere (Meisrimler et al., 2011; Lüthje et al., 2014) and stored at −76°C until use. Total protein extracts from corn roots (12 days) were acquired by grinding with liquid nitrogen, followed by extraction in Tris-HCl buffer pH 7.6 (50 mM NaCl, 1 mM DTT, 1% Triton X-100) for 1 h at 4°C. Extraction was followed by centrifugation at 10,000 g for 10 min (Beckman, Avanti, Germany). All extraction buffers contained protease inhibitors (Sigma Aldrich, France) and phosphatase inhibitors (Sigma Aldrich, France). Protein amounts were quantified as described by Bradford (1976) in the presence of 0.01% Triton X-100 using bovine serum albumin as the standard.

### First dimension–non-reducing IEF and NEPHGE

**Figure 1:**
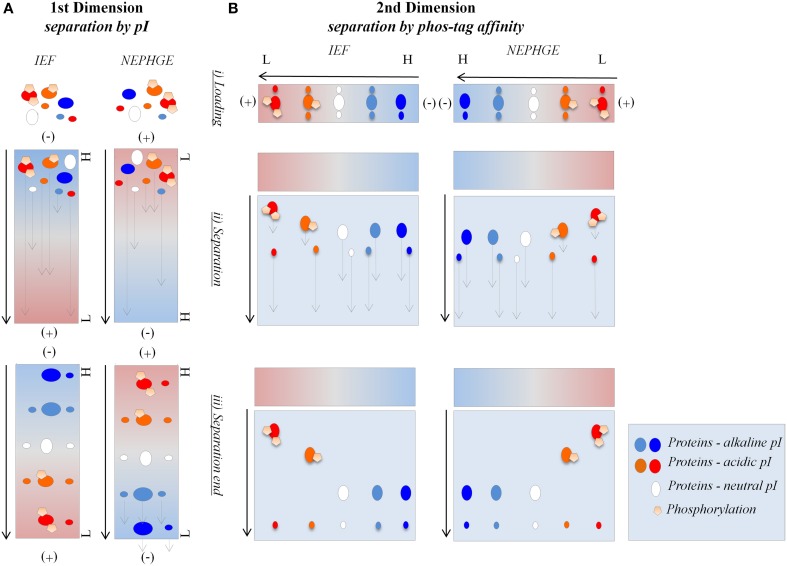
**Separation model of IEF/phos-tag hrCNE and NEPHGE/phos-tag hrCNE. (A)** The buffer system in the IEF consisted of NaOH (upper chamber) and H_3_PO_4_ (lower chamber). Both, buffer system and polarity of IEF were reversed for separation by NEPHGE. Proteins moved through the IEF until they reached their pI within the pH gradient of the gel [alkaline (blue) to acidic (red)]. During NEPHGE, proteins moved in the direction of the cathode due to their pI. NEPHGE was stopped before the pH equilibrium was reached to keep proteins with an alkaline pI in the gel. After separation by pI, gel lanes were sliced out, equilibrated and transferred to the second dimension. **(B)** In the second dimension proteins were separated by hrCNE due to their molecular weight. Phos-tag hrCNE separated proteins based on their affinity to the phos-tag. Cathode and anode are indicated as (−) and (+), respectively. An arrow on the left of the gels indicates the direction of separation. Low (L) and high (H) pH are labeled on the right of the first dimensions. Three steps are indicated for first and second dimension: (i) loading the sample, (ii) separation of proteins by electrophoresis, and (iii) final position of the proteins after stopping the electrophoresis.

Similar gels were used for IEF and NEPHGE. Gels consisted of 4.5% acrylamide, 2% ampholytes pH 3–10 (Serva, Heidelberg, Germany), 4 M urea and 2% CHAPS. Gels were always prepared maximum 24 h before use. Minimum polymerization time was 1.5 h at 34°C. Polymerization was triggered by 0.1% ammoniumpersulfat (APS) and 0.01% N,N,N′,N′-tetramethylethylendiamin (TEMED). Sample buffer was prepared as 4× buffers for both separation methods (IEF, NEPHGE). Samples loaded on the gel contained 1 M urea, 10% glycerol, 0.5% CHAPS and 2% ampholytes. Before samples were applied, a pre-run of the gels was accomplished for 45 min at 30 V with no further restrictions. Electrophoresis conditions were described by Lüthje et al. (2014). For NEPHGE, the polarity and the IEF buffer system was reversed (Figure 1). Electrophoresis conditions for native NEPHGE were constantly set to 450 Vh, to keep highly alkaline proteins in the gel (step 1: 100 V, 150 Vh; step 2: 250 V, 250 Vh; step 3: 500 V, 200 Vh).

### Second dimension–hrCNE and phos-tag hrCNE

Standard hrCNE was casted as a gradient gel of 4–16% as described by Lüthje et al. (2014). The content of the phos-tag hrCNE was similar to the standard hrCNE containing additionally phos-tag (Wako Chemicals GmbH, Neuss, Germany) and Mn(II)Cl_2_. Different phos-tag concentrations (0.5 μM, 1 μM, and 10 μM) were tested using phosvitin as standard protein. The phos-tag: MnCl_2_ ratio was always kept at 1:2 as recommended for phos-tag SDS-PAGE. hrCNE and phos-tag hrCNE were casted as 1 mm continuous gradient gels with an acrylamide concentration of 4–16% (Lüthje et al., 2014). Gel slices from the first dimension non-reducing IEF or NEPHGE were equilibrated in hrCNE equilibration buffer (0.1% Triton X-100, 0.07% DOC, 20% glycerol, 0.001% Ponceau S, 50 mM imidazol, 1 M 6-aminohexanoic acid) for 30 min at room temperature. Cathode buffer (50 mM tricine, 7.5 mM imidazol, 0.05% DOC, 0.05% Triton X-100) and anode buffer (25 mM imidazol-HCl, pH 7.0) were used as described by Wittig et al. (2007). The electrophoresis conditions were 30 min 100 V, 45 min and 10 mA per gel limited to 500 V at 4°C.

### In-gel staining procedures

Proteins were stained by Coomassie Colloidal Blue (CCB) for total protein content (Neuhoff et al., 1988). Native in-gel staining was done for heme and Cu proteins with 3,3′,5,5′-tetramethylbenzidine (TMB) and H_2_O_2_ in Na-acetate buffer, pH 5.0 (Lüthje et al., 2014). For native in-gel staining, gels were documented after 1–5 min of incubation in staining solution. NADH dependent Fe(III)-chelate reductase staining was accomplished using 250 μM ferrozine, 125 μM NADH, and 250 μM Fe(III)-citrate (Holden et al., 1991; Meisrimler et al., 2011). Gels were documented before the staining saturated. 3,3-Diaminobenzidine (DAB) staining was accomplished with 300 μM DAB and 100 μM H_2_O_2_ in 250 mM Na-acetate buffer, pH 5.0. NADH/NBT staining was accomplished for NADH dependent flavin reductase family proteins in Tris-HCl at pH 7.4 (Meisrimler et al., 2011). Scanning of the gels was done with 600 dpi resolution (Epson Photo scanner V 700, Epson, Germany) and files were saved in TIF format.

Pro-Q Diamond® staining for phosphoproteins was accomplished after in-gel activity staining and fixation using the fast staining protocol as recommended by the provider. IEF or NEPHGE gels were fixed in 20% TCA and hrCNE or phos-tag hrCNE were fixed in 40% MeOH and 10% acetic acid overnight. All gels were washed once for 30 min and twice for 10 min in ultrapure water before phospho staining. After destaining gels were washed three times with ultrapure water, followed by detection using a CCD camera at 560 nm (Biorad, Chemdoc, Germany).

At least three independent technical replicates were accomplished per staining in the second dimension to show specificity of the spots in relation to their phosphorylation (Supplemental Datas 1, 2). Students *T*-test was used to statistically test the protein separation shift between hrCNE and phos-tag hrCNE for significance.

### Protein digestion and mass spectrometry

Gel spots were cut out and proteins reduced with DTT, alkylated with iodoacetamide and digested with trypsin by standard protocol described in Meisrimler et al. (2014). After digestion, the gel pieces were repeatedly extracted (50% acetonitrile/5% formic acid) and the combined extracts dried down in a vacuum concentrator.

For QTOF, Premier tandem MS analysis peptide extracts were dried in a vacuum concentrator and resuspended in 20 mL 0.1% formic acid. The samples were centrifuged at 16,000 rpm and 2–4 μL of the digest were used for LC-MS runs which were done on a QTOF Premier tandem mass spectrometer (Waters-Micromass, Eschborn, Germany) equipped with an Aquity UPLC (Waters, Eschborn, Germany). Samples were applied onto a trapping column (Waters nanoAquity UPLC column, C18, 180 μm × 20 mm), washed for 10 min with 5% acetonitrile, 0.1% formic acid (5 μ L/min) and then eluted onto the separation column (Waters nanoAquity UPLC column, C18, 1.7 μm BEH130, 75 μm × 200 mm, 200 nL/min) with a gradient (A, 0.1% formic acid; B, 0.1% formic acid in acetonitrile, 5–50% B in either 60 or 120 min). The spray was done from a silica emitter with a 10 μm tip (PicoTip FS360-20-10, New Objective) at a capillary voltage of 1.5 kV. For data acquisition, the MSE technique was applied: alternating scans (0.95 s, 0.05 s interscan delay) with low (4 eV) and high (ramp from 20 to 35 eV) collision energy was recorded (Silva et al., 2005; Li et al., 2009). The data were evaluated with the software package Protein Lynx Global Server version 2.5.2 (Waters, Eschborn, Germany) searching the Uniprot database and Uniprot tremble (Jan 2014 update). At intervals of 10 s, a lockspray spectrum (1 pmol/μ L [Glu1] Fibrinopeptide B (Sigma)) was recorded. Using lockspray correction, a mass accuracy of <7 ppm was achieved in the MS mode.

Orbitrap measurements were performed in an Orbitrap Fusion Tribrid instrument (LC-ESI-OT-MS, Orbitrap Fusion, Thermo Scientific) equipped with a HPLC (Ultimate 3000, Thermo, LC parameter: RP C18 Column (Acclaim PepMap RSLC, Thermo, 75 μm × 250 mm, 2 μm, 100Å), flow: 0.3 μl/min, solvent A: H_2_O/0.1% formic acid, solvent B: acetonitril/0.1% formic acid, gradient: 2–30% B in 30 min). The Orbitrap was operated with a resolution of 120,000 in positive ion mode. Precursor ions were selected using data dependent acquisition mode (DDA) and fragmented with a normalized HCD (high-energy collision dissociation) energy of 35%. The fragment ions were detected in the linear ion-trap (rapid mode).

The LC-ESI-OT-MS data were processed with Proteome Discoverer v1.4.1.14 (Thermo Scientific) using the following parameters: precursor mass tolerance 10 ppm, fragment mass tolerance 0.2 Da, 1 missed cleavage, carbamidomethylation on Cys as fixed and oxidation on Met and phosphorylation on Ser, The and Tyr as variable modifications. All peptide assignments were verified by manual inspection.

## Results and discussion

### Two-dimensional zymograms

For the separation in the first dimension non-reducing IEF and NEPHGE were accomplished, separating proteins based on their pI. For NEPHGE the pH gradient was directed in the opposite direction (acidic to alkaline) than for IEF (Figure 1). Protein separation by NEPHGE was stopped before the pH equilibrium was reached. Therefore, NEPHGE could not be used to calculate the pI of a protein. NEPHGE is normally used for highly alkaline proteins (e.g., membrane proteins) that otherwise would be lost for any analysis by PAGE and the following MS identification. To reach comparability of NEPHGE replicates, the Vh were kept constant between different gel runs (Lopez, 2002). IEF and NEPHGE could be used to separate the same, differently phosphorylated, enzyme, based on their pI shift (Zhu et al., 2005). The shift is introduced by the extra negative charge of the phosphorylation and was also used for separation of phosphoproteins in IPG-strip/SDS-PAGE (Larsen et al., 2001).

**Figure 2:**
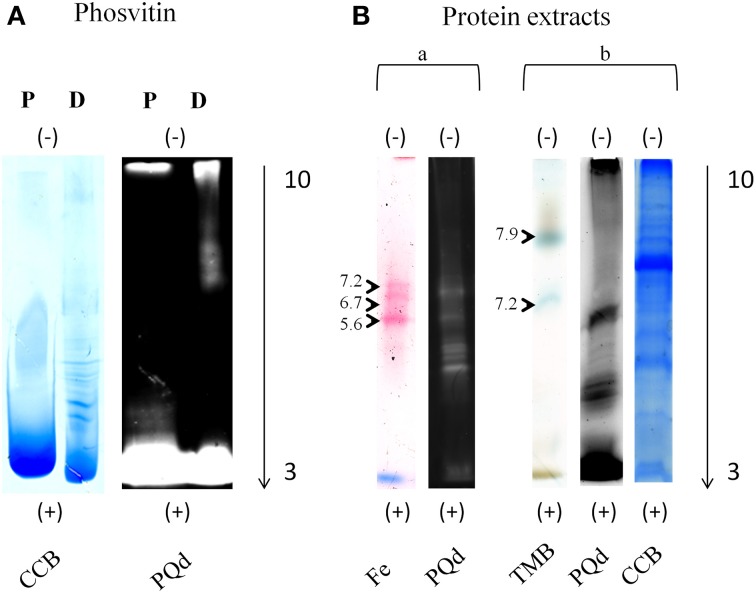
**IEF in-gel activity staining compatibility with Pro-Q Diamond® staining**. Separation of phosvitin and protein extracts (50 μg) in non-reducing IEF pH 3–10. **(A)** Phosvitin separation followed by Pro-Q Diamond® and CCB staining. P, phosphorylated; D, partially dephosphorylated. **(B)** Separation of protein extracts followed by in-gel activity, Pro-Q Diamond®, and CCB staining. (a) Fe, Fe(III)-citrate/ferrozine/NADH (microsomes, pea roots); (b) TMB, TMB/H_2_O_2_ (soluble fraction, corn leaves). Each in-gel activity staining procedure was followed by Pro-Q Diamond® staining in the same gel. Type of staining is indicated at the bottom and pI of the bands indicated on the left of the gels. Cathode and anode are indicated as (−) and (+), respectively. Arrows show the direction of protein separation.

The non-reducing IEF sample buffer contained 1 M urea and only CHAPS as detergent, resulting in a clear resolution in the first dimension of soluble proteins and microsomes (Figure 2). The pre-run before IEF and NEPHGE increased resolution and activity of the bands. Similar effects have been shown for native Tris-PAGEs in the past (Weydert and Cullen, 2010).

Urea can denature proteins because it diminishes the hydrophobic effect by displacing water in the solvation shell and because it specifically binds to amide units. It has been shown that urea interacts differently with different functional groups, resulting in heterogenic effects on the protein activity. Also, effects of urea have been shown to be reversible, if not used directly in an assay. Therefore, inhibitory concentrations of urea on protein activities strongly rely on the type of protein (Rajagopalan et al., 1961; Kim and Woodward, 1993; Zou et al., 1998; Garfin, 2003; Choi et al., 2004). For more urea (denaturing compounds) sensitive proteins, the optimal urea concentration of gels and sample buffers have to be adjusted based on the level of enzyme activity assayed in the urea-containing enzyme reaction buffer.

After separation by pI, gel lanes were sliced out, equilibrated and transferred to the second dimension as described earlier by Lüthje et al. (2014).

One of the most critical points for two-dimensional PAGE was the transfer of the proteins from the first to the second dimension. For the equilibration of the first dimension IEF/NEPHGE, the second dimension hrCNE gel-buffer was supplemented with 0.1% Triton X-100 and 0.07% DOC and gels were equilibrated by continuous shaking at room temperature. This equilibration buffer was applicable for all soluble samples, whereas microsomal fractions showed inferior separation (Supplemental Data 3) due to increased hydrophobicity often observed with membrane protein samples (Meisrimler and Lüthje, 2012). Higher concentrations of detergent had a negative influence on the separation of the proteins and produced irregularities in the separation pattern (data not shown). Sample-dependent adaptions on the presented method are possibly needed for strongly hydrophobic proteins, e.g., testing different detergent combinations, concentrations and solubilization time.

Second dimension standard hrCNE separates proteins based on the size of a protein. In phos-tag hrCNE phosphoproteins were separated by their affinity to the phos-tag under native conditions that has been shown to be highly specific by Kinoshita et al. (2006) and Kinoshita-Kikuta et al. (2007).

In comparison to standard two- dimensional gel electrophoresis (e.g., IPG-strip/SDS-PAGE) the combination of non-reducing IEF/NEPHGE with phos-tag hrCNE excludes the effects of DTT, precipitation and heating. These treatments can affect the activity of a protein and their phosphorylations. In-gel staining like Pro-Q Diamond®, all blue and quercetin, most commonly applied after IPG-strip/SDS-PAGE, only show the current form of phosphoproteins in the gels (Orsatti et al., 2009; Wang et al., 2014). In case of phosphorylation loss before staining, the information would be lost for further analysis. Also, multiple post-translational modifications per protein could affect the pI shift in the first dimension and analysis will be difficult. Phos-tag hrCNE was focused only on phosphoproteins comparable to affinity chromatography, e.g., IMAC (Machida et al., 2007). Other post-translational modifications were excluded as effectors in the second dimension and therefore results were easier to interpret.

Binding abilities and optimal concentrations of the phos-tag in the second dimension hrCNE were tested using phosvitin as a standard for protein phosphorylations (Samaraweera et al., 2011). Alongside, partially dephosphorylated phosvitin was used as a control. First dimension non-reducing IEF confirmed the theoretical pI of 4.5 of phosvitin, showing a pI of 4.4–4.6 for the phosphorylated phosvitin. The partially dephosphorylated protein showed bands with pI of 5.2 and higher (Figure 2A). Combinability of the non-reducing IEF with Pro-Q Diamond® was first tested with phosvitin and was followed for the combination of native in-gel staining followed by Pro-Q Diamond® (Figure 2).

**Figure 3:**
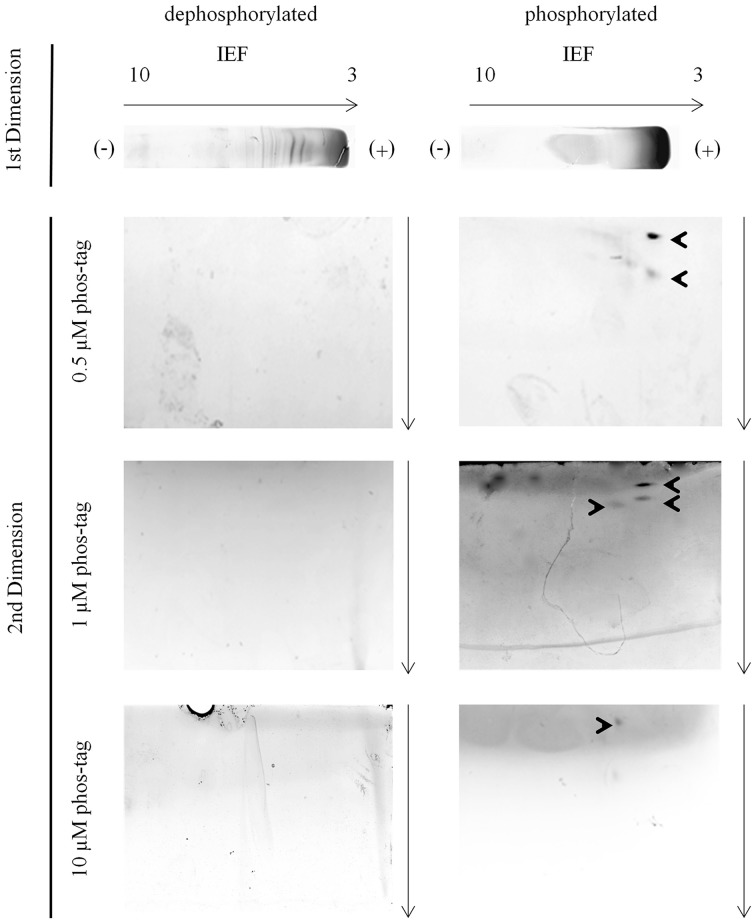
**Phosvitin separation—IEF/hrCNE vs. IEF/phos-tag hrCNE**. Phosvitin (phosphorylated and partially dephosphorylated) was separated in the first dimension non-reducing IEF specified on the top of the figure. hrCNE 4–16% or phos-tag hrCNE 4–16% were accomplished in the second dimension. The indicated phos-tag concentrations (0.5, 1.0 and 10 μM) were chosen based on former results of Deswal et al. (2010). The pH of the IEF (3–10) is indicated on the top of the first dimension. Arrows show the direction of protein separation. Cathode and anode are indicated as (−) and (+), respectively.

In the second dimension phosvitin was observable in the 0.5 μM, 1 μM, and 10 μM phos-tag hrCNE (Figure 3). Phosvitin was not visible in 0.1 μM, similar to the standard hrCNE without phos-tag or the dephosphorylated protein (Figure 3). The concentration of 0.1 μM phos-tag was under the limit of the binding ability for phosphoproteins. Overall, best resolution of the phosvitin was achieved in the 0.5 μM phos-tag hrCNE.

The fact that phosvitin was only detectable in its phosphorylated form in the second dimension was caused by the resolution of the hrCNE. hrCNE is normally used for the separation of native protein complexes which have fairly high molecular masses. Proteins with lower molecular masses than 45 kDa were not found after separation in the hrCNE or move very close to the separation front (Lüthje et al., 2014). Based on this fact, the combination of non-reducing IEF/NEPHGE with hrCNE was most useful for proteins with a size above 50 kDa. This fact was one of the major constraints of the presented method. This restriction could possibly be overcome by exchanging the hrCNE to a native Tris-PAGE (Weydert and Cullen, 2010). This has to be further investigated in the future.

The optimal concentration of 0.5–1 μM phos-tag used in the presented protocol was found to be in the range reported for first dimension BNE (Deswal et al., 2010). However, the needed phos-tag concentration is much lower than in phos-tag SDS-PAGE. Deswal et al. (2010) discussed the difference in the needed phos-tag concentration between first dimension phos-tag BNE and phos-tag SDS-PAGE, speculating that it might be related to the difference in bis-acrylamid to acrylamide ratio used in the two methods. For the presented hrCNE protocol we used a similar bis-acrylamid to acrylamid ratio than used for standard SDS-PAGE, showing that the effect of decreased need of phos-tag was not related to this ratio, but more to the fact that proteins were closer to native conditions. It is highly possible that the treatment of samples with SDS, reducing agents like DTT and heating in the standard protocol affects the phosphorylation sites or the accessibility of the phosphorylation sites that bind to the phos-tag, similar to the treatment before standard IPG-strip/SDS-PAGE.

Phosvitin was not detectable in non-reducing NEPHGE (450 Vh) optimized for highly alkaline proteins. Therefore, phosvitin cannot be used as standard for the pre-separation by non-reducing NEPHGE in the first dimension. An ideal standard for the separation in the alkaline range has still to be found.

NEPHGE protocols found in the literature normally use higher Vh than in the present protocol (Lopez, 2002). Preliminary work with plant samples showed that strong alkaline bands already moved out of the gel for higher Vh (data not shown). Based on these results, separation in NEPHGE was done constantly at 450 Vh to make replicates comparable (Supplemental Data 4).

### Colorimetric staining and identification of proteins

**Figure 4:**
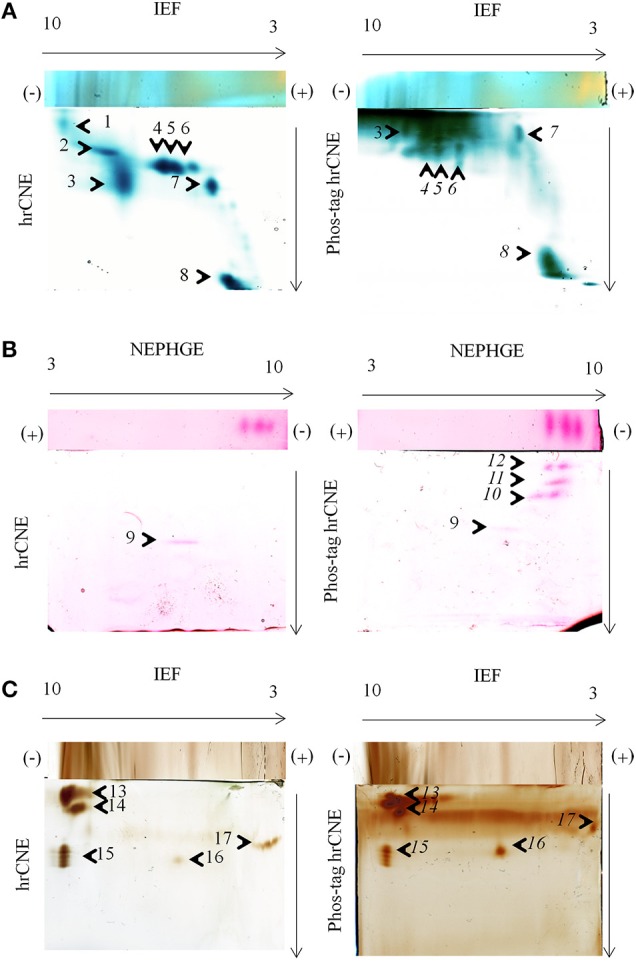
**In-gel activity in two-dimensional zymograms**. Three different in-gel detections were tested for hrCNE 4–16% and phos-tag hrCNE 4–16%. **(A)** TMB/H_2_O_2_ staining of soluble proteins (50 μg) from maize leaves. **(B)** NADH-dependent Fe(III)-reductase staining with ferrozine for soluble proteins (75 μg) of pea roots. **(C)** DAB/H_2_O_2_ staining for total protein extracts (50 μg) from corn roots. Separation type for the first dimension (IEF or NEPHGE) is indicated on top and second dimension on the side of the gels. The pH of the IEF (3–10) is indicated on the top of the first dimension. Arrows show the direction of protein separation. Cathode and anode are indicated as (−) and (+), respectively. Numbered spots were analyzed for the shift on phos-tag hrCNE compared to hrCNE and analyzed by LC-MS/MS, numbers of potentially phosphorylated proteins are written in italic letters.

Functionality of the two dimensional zymograms was tested with soluble proteins from corn leaves, soluble proteins of pea roots and total protein extracts of corn roots (Figure 4). The sample variety showed the independence of the method from the origin and age of a sample.

TMB, DAB, and ferrozine staining were chosen to test the properties of the conventional two dimensional zymograms and the phos-tag zymograms. Before accomplishing staining procedures in the second dimension compatibility with the first dimension non-reducing IEF was tested (Figure 2). Different samples were separated on non-reducing IEF and stained with TMB, ferrozine (Figures 2B, 4), DAB (Figure 4), and NBT (Supplemental Data 5). Detectable bands in TMB and ferrozine staining are indicated with their corresponding pI (Figure 2). All stainings have been published to be specific for different protein groups. TMB/H_2_O_2_ staining has commonly been used for the detection of Fe and Cu containing proteins, e.g., flavocytochromes, peroxidases, blue copper proteins. DAB/H_2_O_2_ has been shown to be specific for oxygen radical producing enzymes, mostly heme-containing proteins, e.g., peroxidases, but not for Cu-containing enzymes (Lüthje et al., 2014). The Fe(III)-reductase staining with ferrozine and NADH is highly specific for enzymes that are able to reduce Fe(III) to Fe(II) at the given pH using NADH as a co-substrate (Holden et al., 1991; Meisrimler et al., 2011). After reduction, the Fe(II) is bound in a stable complex with ferrozine (Viollier et al., 2000). NBT/NADH staining was also accomplished in the first dimension. This staining was published to be specific for NADH using reductases like quinone reductases (Yan and Forster, 2009; Meisrimler et al., 2011). The formazan salt formed in the reaction with NBT was too stable to be removed from the gel and stainings were not compatible with Pro-Q Diamond® staining (Supplemental Data 5).

TMB, DAB and Fe(III)-reductase staining procedures were accomplished in the second dimension with and without phos-tag, proving the functionality of non-reducing IEF/NEPHGE with the second dimension hrCNE and phos-tag hrCNE as zymograms. However, separation in the second dimension appeared to be the most problematic step. Especially the highly sensitive TMB staining for the relatively extensive group of heme and Cu proteins showed higher backgrounds (Figure 4B). The phos-tag hrCNE exhibited the highest background, making it difficult to analyze gels.

Migration of proteins was compared for hrCNE and phos-tag hrCNE. The phosphorylation of a protein causes a slower migration in phos-tag hrCNE due to their affinity to the phos-tag, leading to a measurable shift between hrCNE and phos-tag hrCNE. However, six spots (9, 13–16) showed no significant shift on the phos-tag hrCNE, when compared to the hrCNE. These proteins had no affinity for the phos-tag and were not phosphorylated. For spot 1 and 2, the shift was not computable due to the high background in the top of the phos-tag hrCNE gel. All other spots (4–8, 10–12) had a significant migration shift of more than 10% of the total migration distance (gel length).

The main spots, showing a significant shift on the phos-tag hrCNE compared to the hrCNE with a clear appearance on both gels, were picked and identified by LC-MS (Table 1). Spots 4–6 were identified as fructose bisphosphate aldolase on both gels. Spot 8 was identified as fruit protein (B4FRC8) and as an uncharacterized protein on the phos-tag hrCNE (Table 1; Supplemental Table 1). Based on the small amount of peptides detectable it was not possible to detect specific phosphopeptides in the analyzed spots. The fruit protein was identified also in a former phosphoproteome study available at http://www.ebi.ac.uk/pride/archive/projects/PRD000721 (Bonhomme et al., 2012). Spot 8 was additionally analyzed using LC-MS Orbitrap. Further proteins were significantly identified but not all were related to the TMB staining (Supplemental Table 2).

**Table 1:** **Identified Proteins**.

**No**	**Protein name**	**ID**	**Shift %**	**P**	**Pept**	**pI**	**MW (kD)**	**NetPhos**	**Literature**
1	n.i.	–	?	?	–	–	–	–	n.a.
2	n.i.	–	?	?	–	–	–	–	n.a.
3	n.i.	–	24^*^	+	–	–	–	–	n.a.
4	Fructose bisphosphatealdolase	Q40677	18^**^	+	2	6.4	42	Ser: 8 Thr: 2 Tyr: 4	n.a.
5	Fructose bisphosphatealdolase	C0PD30	18^**^	+	6	6.4	38	Ser: 8 Thr: 3 Tyr: 4	n.a.
6	Fructose bisphosphatealdolase 2	Q944G9	18^**^	+	10	7.1	43	Ser: 13 Thr: 5 Tyr: 2	Aryal et al., 2012
7	n.i.	–	32^*^	+	–	–	–	–	n.a.
8	Full Fruit protein	B6TA31	13^*^	+	11	7.0	32	Ser: 12 Thr: 2 Tyr: 3	Bonhomme et al., 2012
	Full uncharacterized protein	M0XGV2		+	2	4.8	32	Ser: 7 Thr: 4 Tyr: 4	n.a.
9	6,7-dimethyl 8-ribityllumazine synthase	At2g44050	0.3^*^	–	2^*^	8.6	–	Ser: 12 Thr: 3 Tyr: 1	n.a.
10	n.i.	–	100^**^	+	–	–	–	–	n.a.
11	Ferritin	F4JD24	100^**^	+	1^*^	8.7	24	Ser: 16 Thr: 6 Tyr: 6	Beazley et al., 2009
12	n.i.	–	100^**^	+	–	–	–	–	n.a.
13	n.i.	–	4^*^	–	–	–	–	–	n.a.
14	n.i.	–	1^*^	–	–	–	–	–	n.a.
15	Peroxidase 42	A5H453	2^**^	–	3	5.8	32	Ser: 13 Thr: 4 Tyr: 4	n.a.
16	Peroxidase 66	A5H454	4^*^	–	3	8.0	33	Ser: 13 Thr: 3 Tyr: 4	n.a.
17	n.i.	–	4^*^	–	–	–	–	–	n.a.

Protein spots picked from the second dimension hrCNE and phos-tag hrCNE (Figure 4) were digested by trypsin and analyzed by MS. Protein names listed in UniProt are used in the table. No., spot number; ID, accession number in UniProt (http://www.uniprot.org/); Shift, difference in the separation distance of the spot on phos-tag hrCNE and hrCNE in % (?, if unclear). Significant changes (student's T-test) were indicated by

* ≤ 0.01 and

** ≤ 0.001 for n = 3 technical replicates;

P, phosphorylated form detected (+, positive; −, negative); Pept, number of identified peptides—manual sequencing was marked with an asterisk; pI, theoretical isoelectrical point; MW, theoretical molecular weight in kD; NetPhos, theoretical phosphorylation sites calculated with NetPhos 2.0 (http://www.cbs.dtu.dk/services/NetPhos/); Literature, available literature on phosphorylations of the identified protein (if not available, n.a.).

Phosphorylation sites were verified by *in-silico* analysis for all proteins identified (Table 1). Over all, MS based identification after zymograms is often the biggest challenge. The low abundance of proteins stained in zymograms is based on the high sensitivity of these staining methods [e.g., Fe(III)-reductase or TMB staining] which is often higher than for silver staining. If primary MS results enable good protein identification, phosphopeptide enrichment is recommendable in a second MS analysis to verify the results from the phos-tag zymograms (Dunn et al., 2010). In contrast to the TMB staining, specific protein activities like the NADH-dependent Fe(III) reduction and the DAB staining led to a clear separation of proteins (Figure 4) but were more problematic for protein identification. The protocol for non-reducing IEF or NEPHGE/hrCNE presented is also the first functional protocol for Fe(III)-reductase detection in the second dimension. This staining was only published for the first dimension IEF to date (Holden et al., 1991; Meisrimler et al., 2011). Fe(III)-reductase activity was really sensitive and samples had to be treated carefully, avoiding multiple freeze thawing cycles. For spot 11 on the ferrozine stained phos-tag hrCNE only the peptide ISEYVTQLR was identified for ferritin, which is possibly regulated by phosphorylation under different physiological conditions (Beazley et al., 2009). Ferritin has also a Fe-oxidoreductase function, therefore it is potentially stainable by the ferrozine method. Overall, spots 10–12 were only detectable in the phos-tag hrCNE but not in the hrCNE. Therefore, the calculated shift of these spots was 100%. Detected proteins might have been of small size and migrated close to the front in the hrCNE. In phos-tag hrCNE, they have a strong affinity to the phos-tag and migrate slower. To understand the migration of the ferrozine spots further investigations are needed.

Interestingly, ferrozine activity detected in the soluble fraction was exclusively detectable in the alkaline pH using NEPHGE (Supplemental Data 4), whereas in microsomal fractions it was only detectable at more acidic pH with the pI 5.6, 6.7, and 7.2 (Figure 2). The band with a pI of 5.6 was identified as quinone reductase family protein NP_194457 with the peptide AFLDATGGLWR (sequence coverage 5%, score of 26) by manual sequencing.

Spot 9 was identified as 6,7-dimethyl 8-ribityllumazine synthase with two peptides (FNEIITRPLLEGAVATFK and GAEAALTAIEMASLFEHHLK) that has no activity correlated to the Fe-reduction stained in the gel. In both cases, spot 5 and 6, found peptides were verified manually but final scores were to low for significant identification. Overall, multiple proteins per spot can be a problem for the identification of low abundant proteins responsible for an activity detected in zymograms and MS data have to be handled critically.

Spot 15 and 16 were identified on the DAB stained hrCNE as peroxidases. For all the DAB stained spots no significant shift appeared when the sample was separated by phos-tag hrCNE compared to the hrCNE. The identified peroxidases were part of the class III peroxidases, which have not been shown to be the aim of phosphorylation events. Especially, peroxidases of the excretory pathway seem not to be regulated by phosphorylation possibly due to the lack of excreted kinases and phosphatases.

If a specific protein with known activity has to be analyzed proteins can be pre-separated by chromatography (e.g., affinity, ion exchange). Phosphoprotein enrichment is another option and different variations of the technique are available (different IMACS, phosphoprotein enrichment kits). For plant samples IMAC was successfully applied (Tang et al., 2008), but the protocol might need adaption, as application was not relying on activity preservation.

Furthermore, non-reducing IEF/NEPHGE were stained with Pro-Q Diamond® directly after in-gel activity for ferrozine, TMB, and NBT, resulting in a low amount of phosphoproteins detected in the IEF. No phosphoproteins were detected in NEPHGE, which is possibly due to the high alkaline pH (ampholytes) (Figure 2). Pro-Q Diamond® was also applied in the second dimension after in-gel staining (ferrozine). A few spots were detectable in the standard hrCNE but in phos-tag hrCNE no signal could be found at all (Supplemental Data 3). The reason for the incompatibility is not clear but the phos-tag possibly blocks the phosphorylation site for the staining.

In any case, to identify detected proteins, spots should be picked and analyzed by MS and/or by Western blot. Other specific staining methods are available for different enzyme activities (e.g., malate dehydrogenase, lipoxygenase, superoxide dismutase and others) (Manchenko, 2002). Combination of native two-dimensional gel electrophoresis with the phos-tag technology and the use of specific activity stainings has the benefit that changes of these activities by phosphorylation can be directly monitored. Applications of the method can be the observation of specific activities by phosphorylation under differential stress conditions (Supplemental Data 6). The method itself should not be used as a stand-alone technique but together with Western blot, MS and specific point mutation of phosphorylation sites it can be used for a dynamic analysis of reactions to stress factors.

## Concluding remarks

In the past years, various MS based approaches have been developed to identify phosphorylated peptides and proteins. In several techniques, phos-tag related molecules were used for the enrichment of phosphorylated peptides. In contrast to MS methods, phos-tag gels can easily be performed using general gel electrophoresis equipment and radioactivity is avoided. Furthermore, all phosphorylations can be detected. Different phosphorylated forms of the same protein can be distinguished. The combination of phos-tag with zymograms allows estimation of the effects of phosphorylation on protein activity. This allows following activation of proteins by phosphorylation and dephosphorylation. The combination with native IEF for low alkaline to acidic proteins and NEPHGE for highly alkaline proteins is helpful to separate proteins by pI, resulting in a higher resolution of different iso-enzymes. Phos-tag gels were not compatible with Pro-Q Diamond®. Protein identification is possible by MS and results can be confirmed by Western blot. In some cases phosphoprotein enrichment by IMAC or alternative might be needed before phos-tag zymograms to get better identifications by MS.

### Conflict of interest statement

The authors declare that the research was conducted in the absence of any commercial or financial relationships that could be construed as a potential conflict of interest.

## Abbreviations

BNE: blue native electrophoresis
CHAPS: 3-[(3-cholamidopropyl)dimethylammonio]-1-propanesulfonate
CNE: clear native electrophoresis
DOC: sodium deoxycholate
DOMA: n-Dodecyl-beta-D-Maltoside
hrCNE: high resolution clear native electrophoresis
IEF: isoelectric focusing
NEPHGE: non-equilibrium pH gel electrophoresis
TMB: 3,3′,5,5′-Tetramethylbenzidine.
