# Supplementary material for: Two-dimensional phos-tag zymograms for tracing phosphoproteins by activity in-gel staining
Source: Front Plant Sci. 2015 Apr 14;6:230. doi: 10.3389/fpls.2015.00230 (PMC4396385; doi:10.3389/fpls.2015.00230)
Supplement: Supplementary file 3 [file Presentation2.PDF]

Supplemental data 1

A) TMB staining (three technical replicates). 50 µg of soluble proteins of corn leaves were applied to the first dimension IEF pH 3-10. Separation in the second dimension was accomplished on hrCNE or phos-tag hrCNE as indicated at the bottom of the gels.

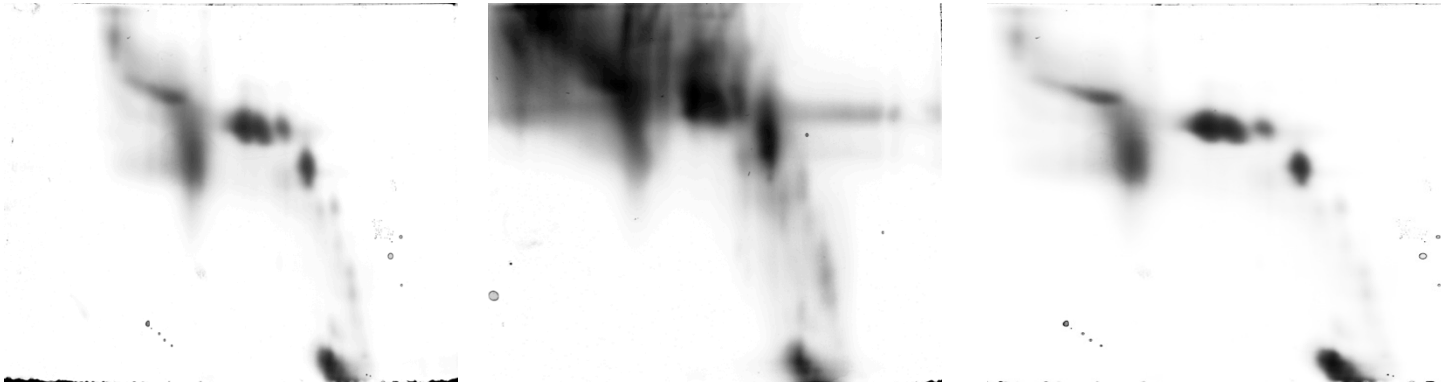

IEF/ hrCNE 4-16%

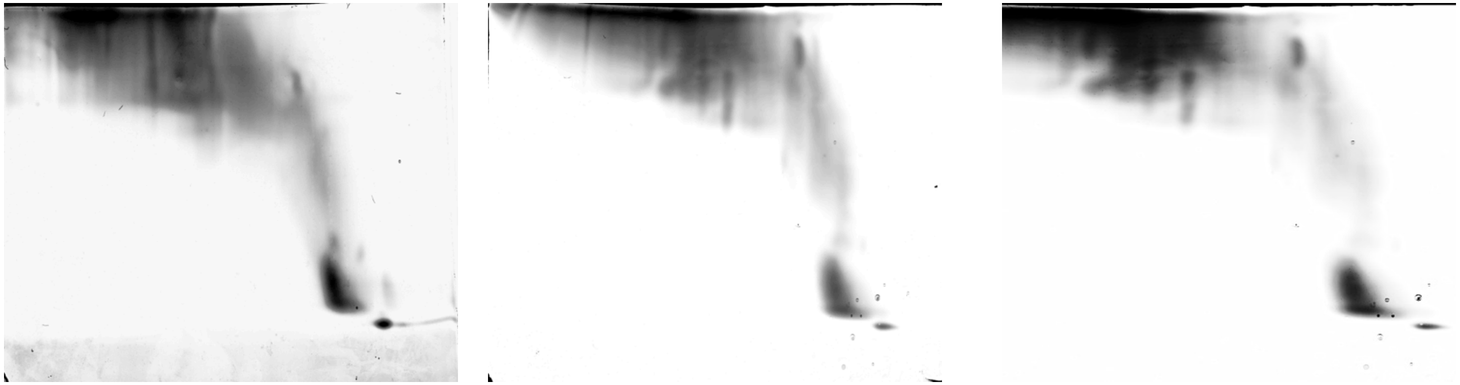

IEF/ phos-tag hrCNE 4-16%

Supplemental data 1

B) Ferrozine staining (independent technical replicates). 75 µg of soluble proteins of pea roots were applied to the first dimension NEPHGE and separated for 450 Vh. Separation in the second dimension was accomplished on hrCNE or phos-tag hrCNE as indicated at the bottom of the gels.

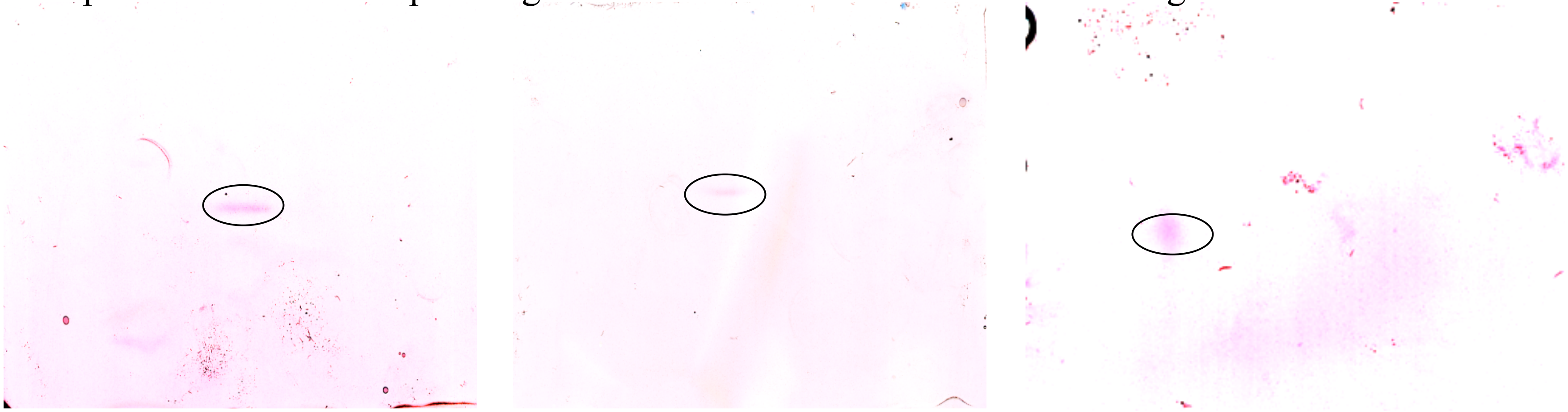

Nephge 450 Vh/ hrCNE 4-16%

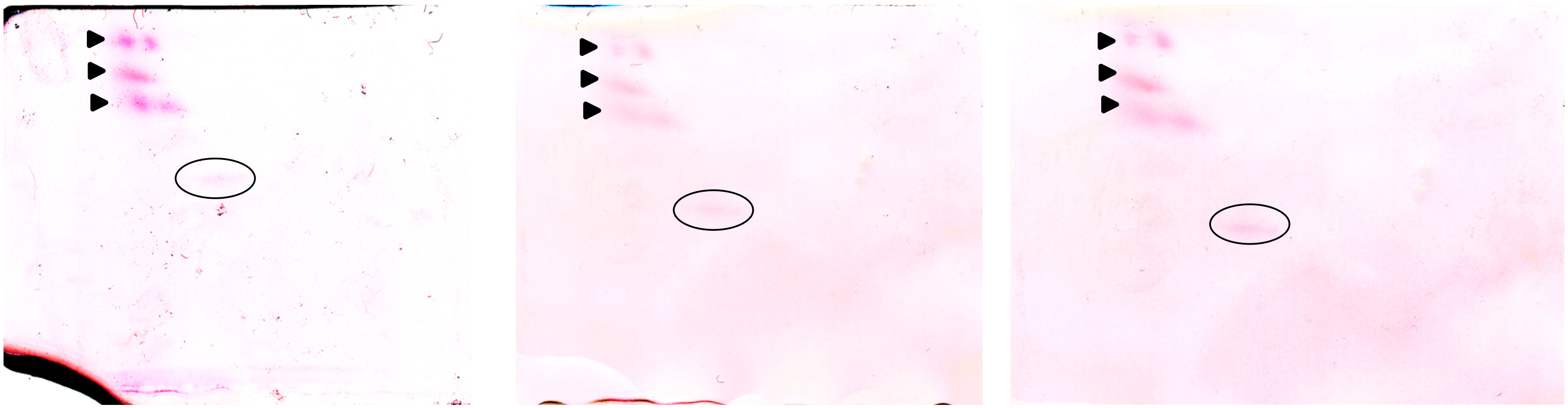

Nephge 450 Vh/ phos-tag hrCNE 4-16%

Supplemental data 1

C) DAB staining (three technical replicates). 50 µg of total protein extract of corn roots were applied to the first dimension IEF pH 3-10. Separation in the second dimension was accomplished on hrCNE or phos-tag hrCNE as indicated at the bottom of the gels.

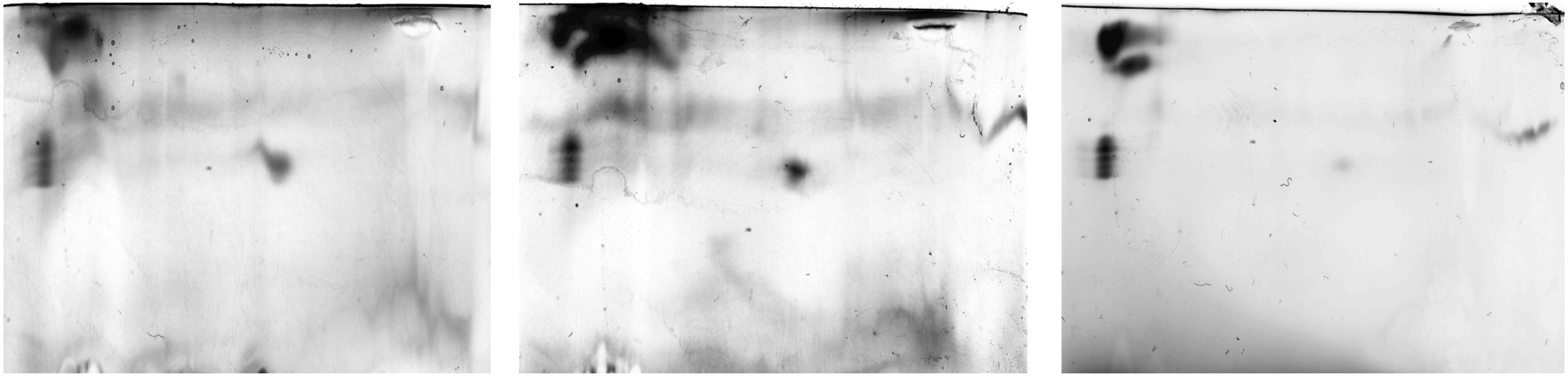

IEF/ hrCNE 4-16%

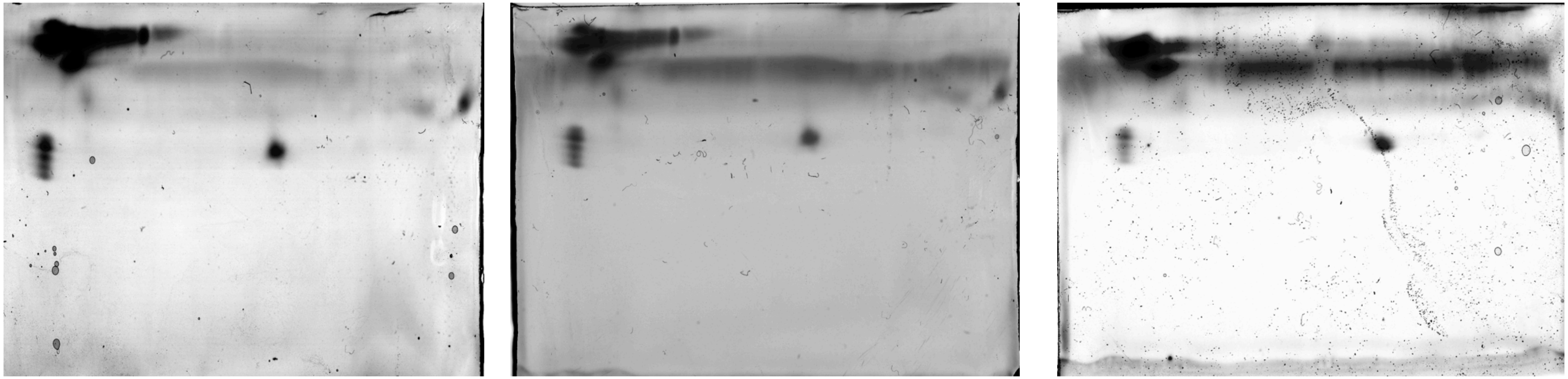

IEF/ phos-tag hrCNE 4-16%

Supplemental data 2

Technical replicates of phos-tag hrCNE for phosvitin. dephos = partially dephosphorylated; phos = untreated phosvitin. 5 µg protein were applied to each first dimension.

0.1 µM phos-tag hrCNE 4-16% 0.5 µM phos-tag hrCNE 4-16%

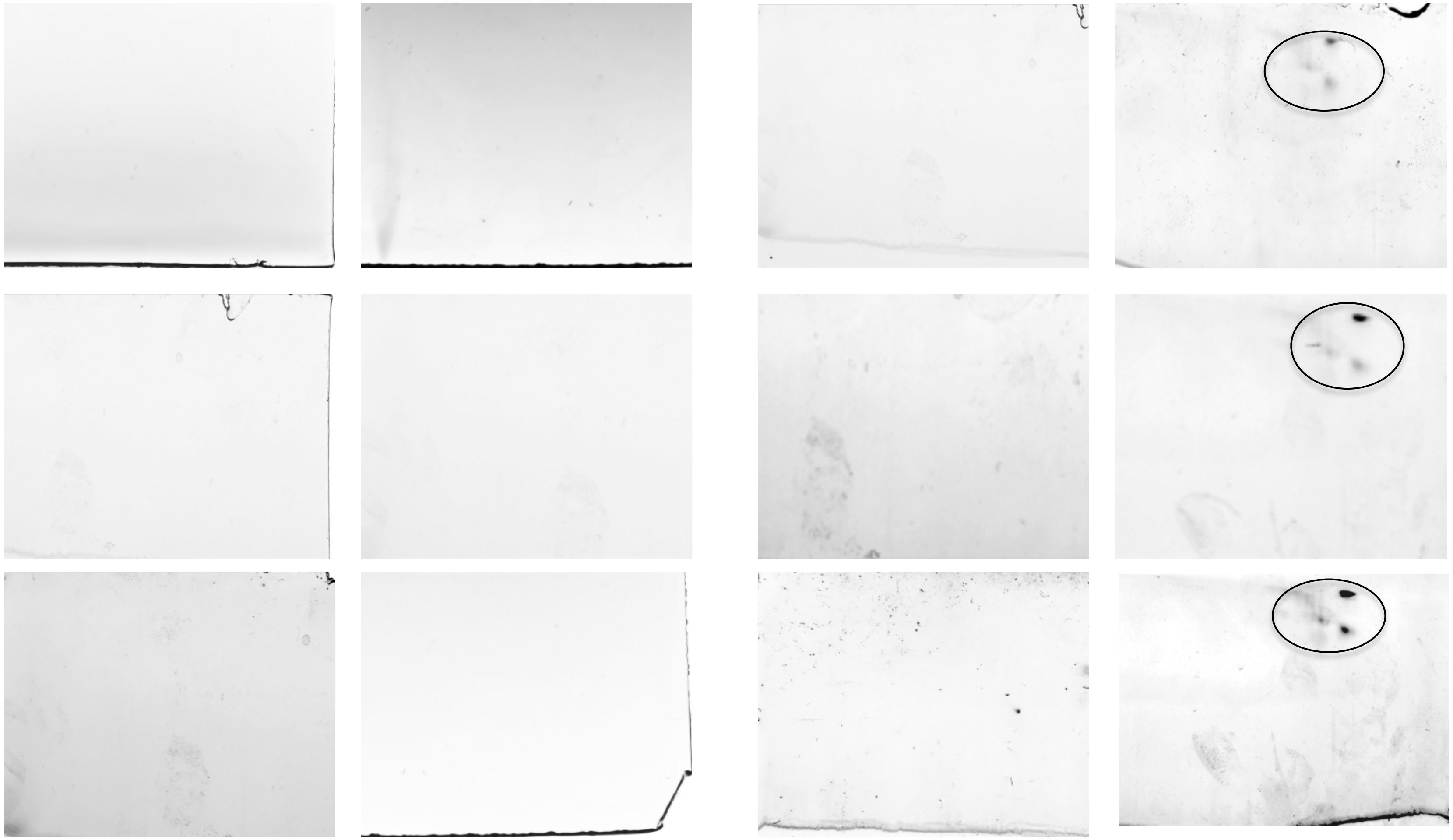

dephos phos dephos phos

Supplemental data 2

Technical replicates of phos-tag hrCNE for phosvitin. dephos = partially dephosphorylated; phos = untreated phosvitin. 5 µg protein were applied to each first dimension.

1 µM phos-tag hrCNE 4-16% | 10 µM phos-tag hrCNE 4-16%

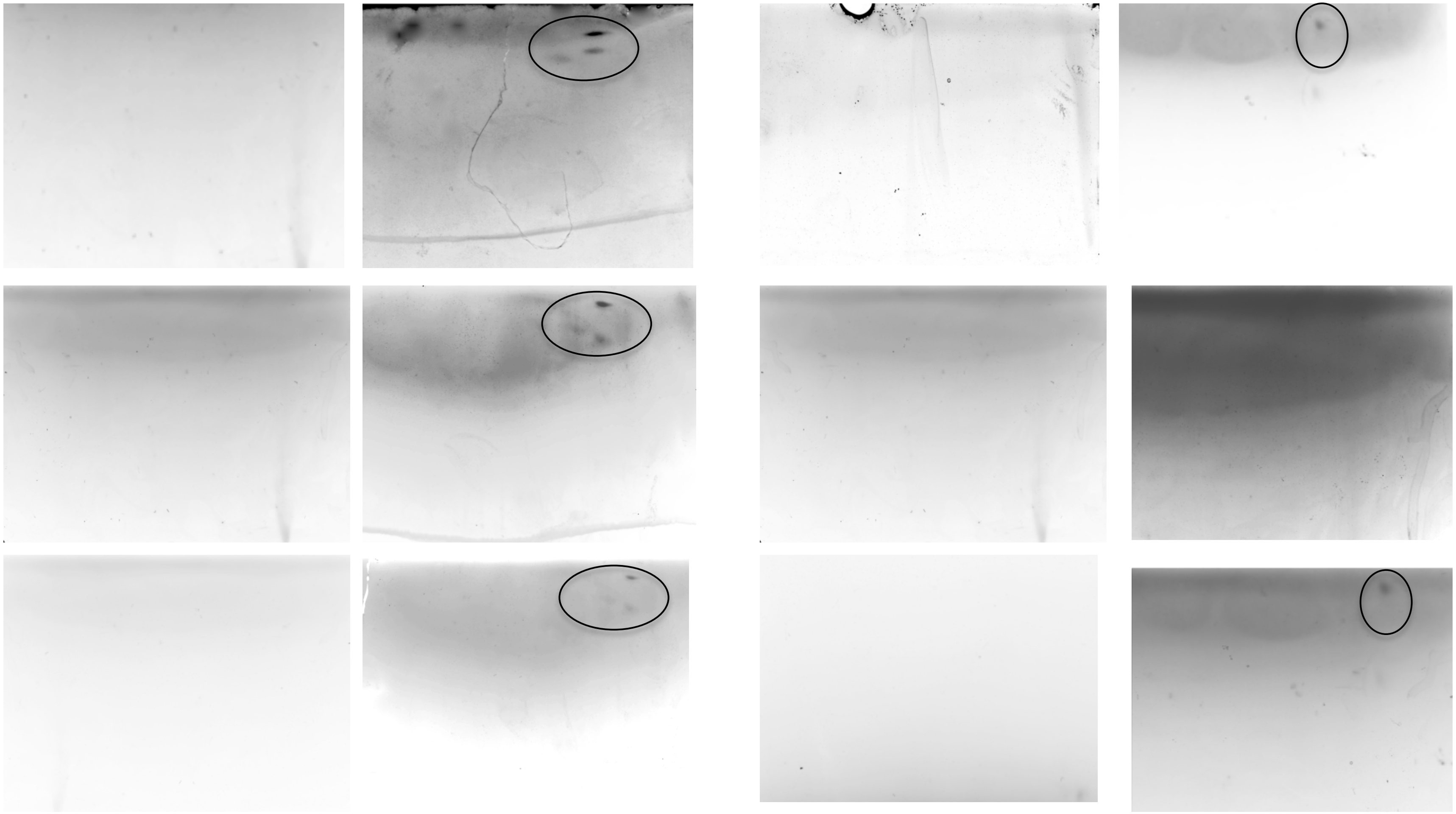

dephos | phos | dephos | phos

Supplemental data 3

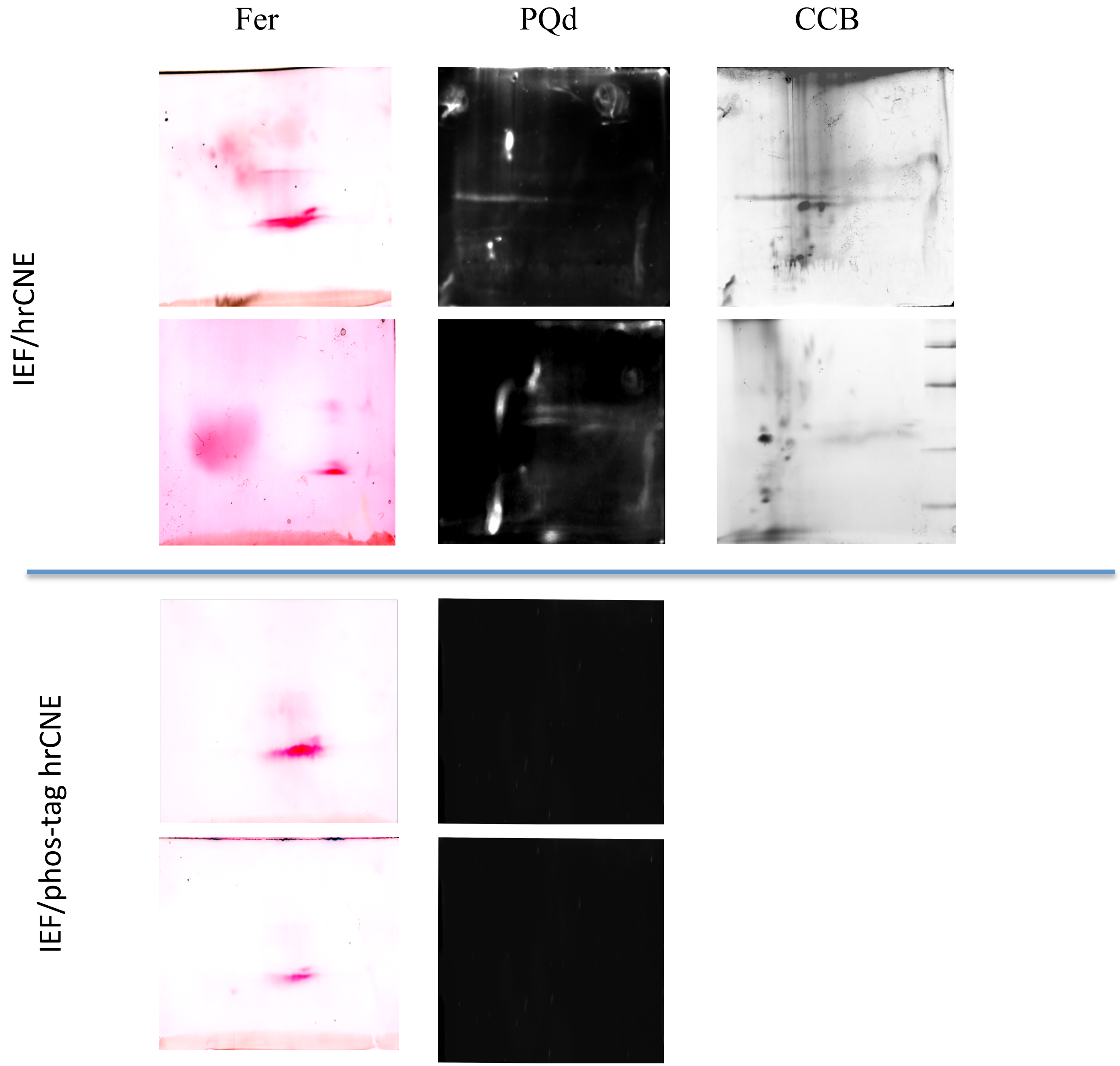

75 µg protein of pea root microsomes were applied to the first dimension IEF pH 3-10. Fer staining was followed by PQd and CCB. The staining accomplished was indicated on the top of the gels. Fer = ferrozine, PQd = Pro-Q Diamond® , CCB = Coomassie Colloidal Blue. Further details can be found in the Material and Method part.

Supplemental data 4

Ferrozine & PQd staining of soluble proteins from pea roots after separation in the first dimension IEF or NEPHGE. PQd staining was accomplished after ferrozine staining of NEPHGE.

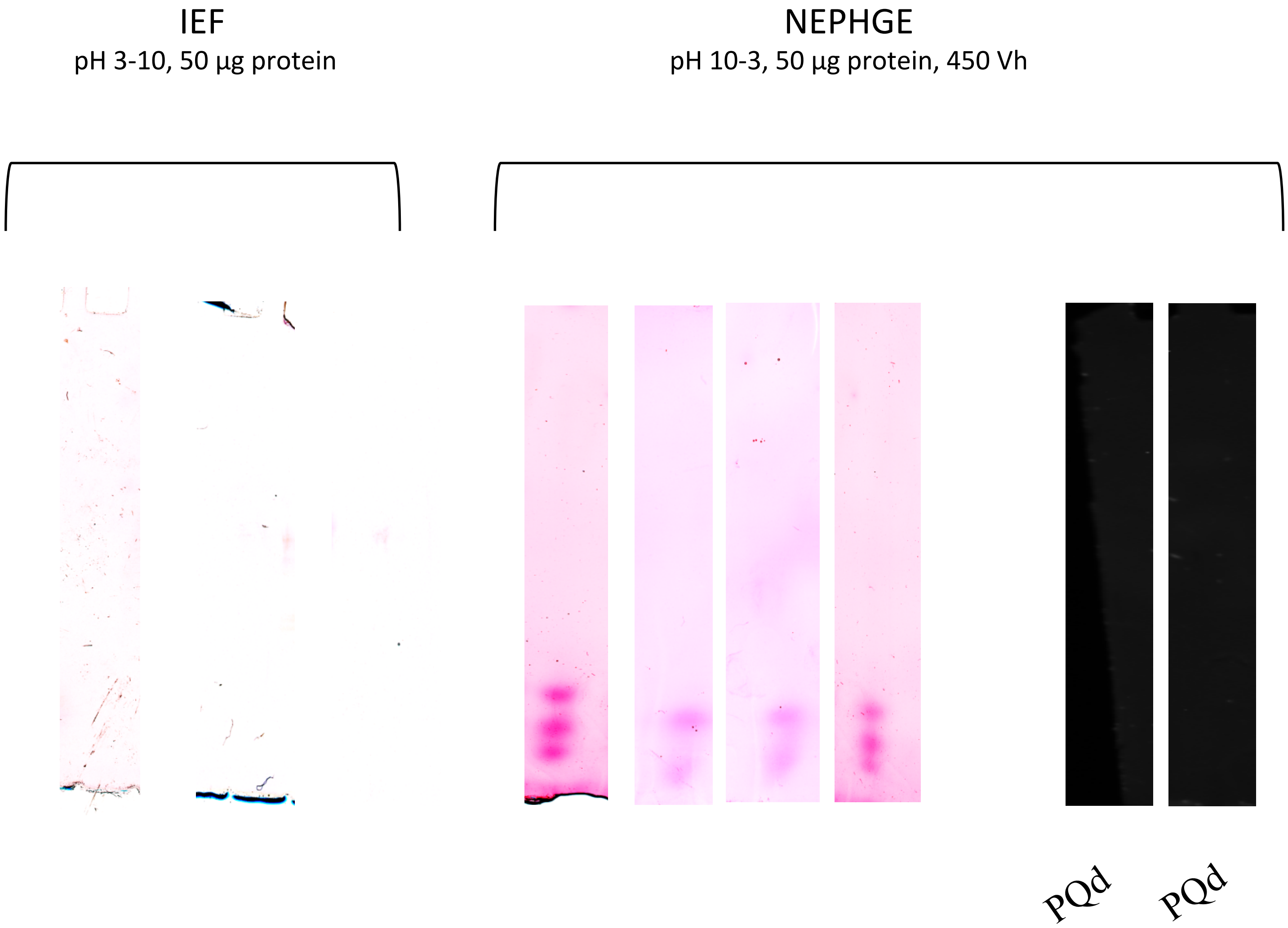

Supplemental data 5

Two independent technical replicates - Compatibility of ferrozine staining and NBT staining with ProQ diamond staining (PQd); samples: microsomes from pea roots

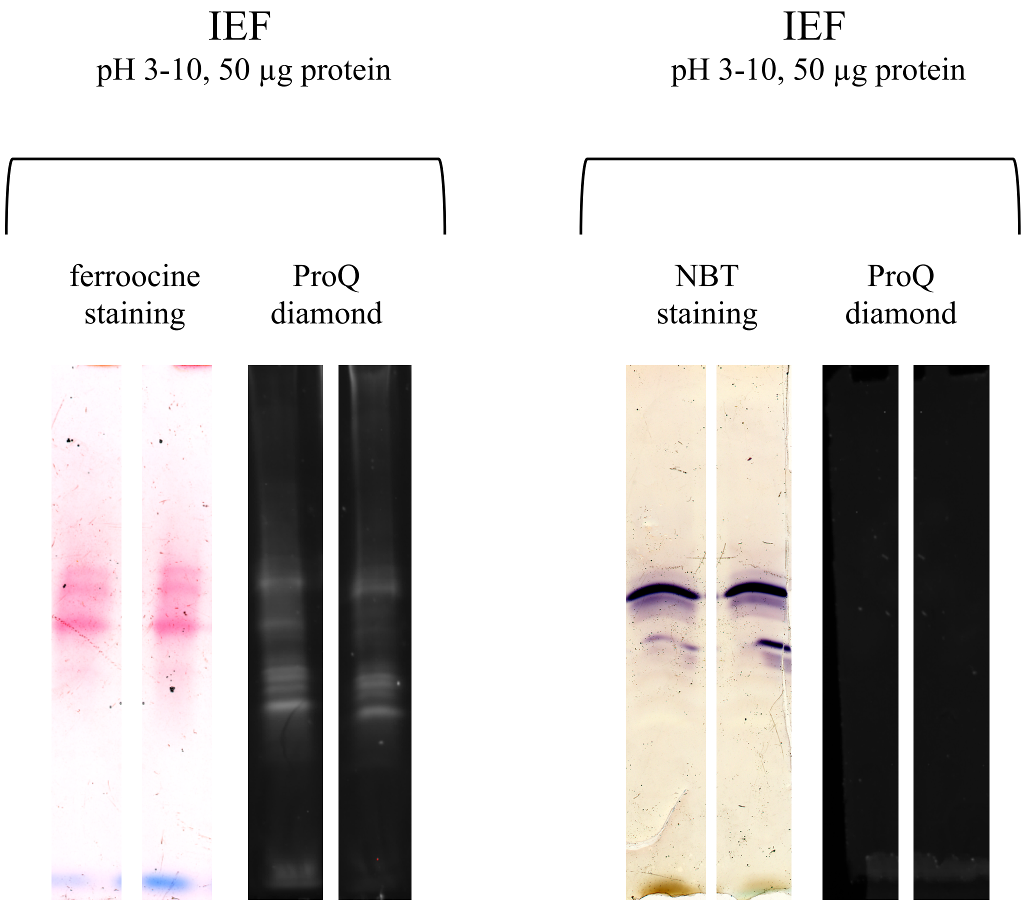

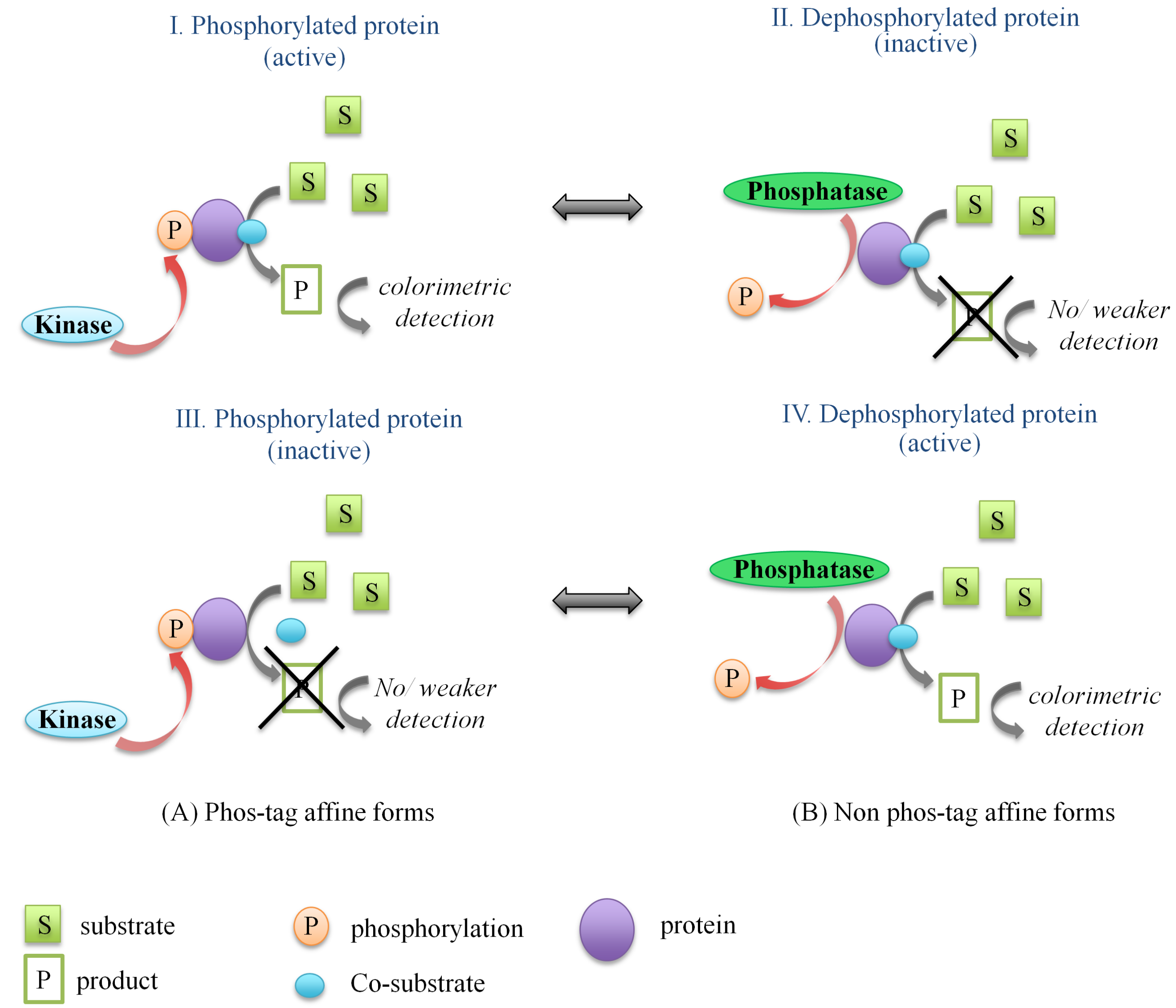

Supplemental data 6: Model for activation and deactivation by phosphorylation

Four options for the effect of phosphorylation on proteins are shown: activation by phosphorylation (I. and II.) and deactivation by phosphorylation (III. and IV.). In both cases it is possible, that the protein activity is only increased or decreased in comparison to the phosphorylated or un-phosphorylated form and not completely activated or deactivated, therefore the conversion rate of the protein is affected. Phosphorylated proteins show higher affinity to the phos-tag and migrate slower in the phos-tag hrCNE (A), whereas non-phosphorylated proteins migrate faster (B). Active proteins can be detected by colorimetric reactions in the phos-tag hrCNE (I. and III.). Physiological aspects by activation and deactivation of a protein and their dependency on phosphorylation can be observed using the combination of phos-tag affinity and activity staining.
